# Does Housing Status Matter in Emergency Medical Services Administration of Naloxone? A Prehospital Cross-sectional Study

**DOI:** 10.5811/westjem.60237

**Published:** 2023-08-28

**Authors:** Tiffany M. Abramson, Corey M. Abramson, Elizabeth Burner, Marc Eckstein, Stephen Sanko, Suzanne Wenzel

**Affiliations:** * University of Southern California, Keck School of Medicine, Department of Emergency Medicine, Divisions of Emergency Medical Services and Research, Los Angeles, California; † University of Arizona, School of Sociology, Tucson, Arizona; ‡ University of Southern California, Suzanne Dworak-Peck School of Social Work, Los Angeles, California

## Abstract

**Introduction:**

Persons experiencing homelessness (PEH) use emergency medical services (EMS) at disproportionately high rates relative to housed individuals due to several factors including disparate access to healthcare. Limited access to care is compounded by higher rates of substance use in PEH. Despite growing attention to the opioid epidemic and housing crisis, differences in EMS naloxone administration by housing status has not been systematically examined. Our objective in this study was to describe EMS administration of naloxone by housing status in the City of Los Angeles.

**Methods:**

This was a 12-month retrospective, cross-sectional analysis of electronic patient care reports (ePCRs) for all 9–1–1 EMS incidents attended by the Los Angeles Fire Department (LAFD), the sole EMS agency for the City of Los Angeles during the study period, January-December 2018. During this time, the City had a population of 3,949,776 with an estimated 31,825 (0.8%) PEH. We included in the study individuals to whom LAFD responders had administered naloxone. Housing status is a mandatory field on ePCRs. The primary study outcome was the incidence of EMS naloxone administration by housing status. We used descriptive statistics and logistic regression models to examine patterns by key covariates.

**Results:**

There were 345,190 EMS incidents during the study period. Naloxone was administered during 2,428 incidents. Of those incidents 608 (25%) involved PEH, and 1,820 (75%) involved housed individuals. Naloxone administration occurred at a rate of 19 per 1,000 PEH, roughly 44 times the rate of housed individuals. A logistic regression model showed that PEH remained 2.38 times more likely to receive naloxone than their housed counterparts, after adjusting for gender, age, and respiratory depression (odds ratio 2.38, 95% confidence interval 2.15–2.64). The most common impressions recorded by the EMS responders who administered naloxone were the same for both groups: overdose; altered level of consciousness; and cardiac arrest. Persons experiencing homelessness who received naloxone were more likely to be male (82% vs 67%) and younger (41.4 vs 46.2 years) than housed individuals.

**Conclusion:**

In the City of Los Angeles, PEH are more likely to receive EMS-administered naloxone than their housed peers even after adjusting for other factors. Future research is needed to understand outcomes and improve care pathways for patients confronting homelessness and opioid use.

Population Health Research Capsule What do we already know about this issue?

*Persons experiencing homelessness (PEH) have higher rates of chronic medical conditions and are disproportionately represented among opioid overdose deaths.*

What was the research question?

*Does the prevalence of naloxone administration by emergency medical services (EMS) vary by housing status?*

What was the major finding of the study?

*Naloxone was administered at a higher rate to PEH (19 vs 0.4/1000). The adjusted OR of naloxone administration was 2.38 times than that of housed peers (95% CI 2.15–2.64).*

How does this improve population health?

*These findings can help drive EMS education and field interventions and identify a target for community risk reduction in this vulnerable population.*

## INTRODUCTION

### Background

Opioid overdoses have reached epidemic proportions in the United States (US), and overdose deaths continue to increase.^1^^,^^2^ Opioid overdose is now among the leading causes of accidental deaths.^3^ The incidence of overdose deaths has increased with the introduction of fentanyl and other synthetic opioids and the aftermath of the COVID-19 pandemic.^1^^,^^2^^,^^4^^–^^6^ Although opioid use disorders (OUD) and other substance use disorders (SUD) affect individuals of all socioeconomic statuses, persons experiencing homelessness (PEH) are at particular risk.^7^^–^^9^ In 2021, 9% of all opioid overdose-related deaths were among PEH.^10^

The housing crisis is another public health epidemic facing the US; it has contributed to a rapidly growing population of PEH with more than 1.5 million individuals experiencing homelessness each year.^11^^,^^12^ Los Angeles County, which has one of the highest housing costs and the second largest population of PEH nationally, is no exception.

Persons experiencing homelessness have higher rates of chronic medical conditions, substance abuse, and psychiatric diagnoses, as well as an overall increase in morbidity and mortality.^13^^–^^16^ Drug overdoses, specifically those associated with opioids, are a common cause of death in PEH.^16^^–^^18^ In one Boston-based study, drug overdose was the leading cause of death and was responsible for one in three deaths in adults experiencing homelessness under the age of 45.^17^ Further, PEH are less likely to have a regular source of medical care and have increased emergency department (ED) utilization and engagement with emergency medical services (EMS).^19^

Persons experiencing homelessness use EMS at disproportionally high rates compared to their housed counterparts. Prior research found that PEH call EMS at a rate 14 times that of their housed counterparts.^20^ At the same time, EMS calls for opioid overdose appear to be on the rise with naloxone administration occurring on almost half a million EMS runs over a two-year period.^21^ As the housing crisis and opioid epidemic collide, it is important to describe how housing status affects EMS utilization and prehospital care for presumed opioid overdose. These findings may lead to recognition of bias in care, identification of opportunities for interventions for those with OUD and limited access to care, and improvement in EMS responders’ education.

### Importance

Despite growing attention to the opioid epidemic and housing crisis, differences in use of 9–1–1 EMS resources for treatment of presumed opioid overdose by PEH and subsequent treatment by EMS has not been described.

### Goals of this Investigation

The primary outcome of interest in this study was how the prevalence of EMS administration of naloxone varies by housing status in the City of Los Angeles. This has important implications for understanding and addressing public health disparities at the intersection of housing, opioids, and poverty.

## METHODS

### Study Design

This was a 12-month retrospective, cross-sectional analysis of electronic health records (EHR) for all 9–1–1 EMS incidents attended by the Los Angeles Fire Department (LAFD) from January 1–December 31, 2018. Study design and reporting adhered to best practices per Strengthening the Reporting of Observational Studies in Epidemiology (STROBE) and Reporting of Studies Conducted using observational routinely collected health data (RECORD) statements.^22^^,^^23^

### Study Setting

The LAFD is the sole entity providing 9–1–1 EMS responses for the City of Los Angeles, the second most populous city in the US. The LAFD receives more than one million 9–1–1 calls and responds to almost 400,000 EMS incidents annually. The City of Los Angeles spans 480 square miles and has 3,949,776 inhabitants, with a homeless population of 31,285 (0.8%).^24^^,^^25^

The LAFD provides EMS care under the guidance of the LA County EMS Agency and its treatment protocols. At the time of the study, the treatment protocol for “overdose/poisoning/ingestion” included intranasal, intramuscular or intravenous naloxone administration for suspected opioid overdose with altered mental status and hypoventilation/apnea.^26^

### Selection Criteria

We included all 9–1–1 EMS calls that resulted in a unique incident number and a completed electronic patient care report (ePCR) with documentation of EMS-administered naloxone during the study period. The LAFD has been using the same ePCR and EHR system (HealthEMS, Stryker, Redmond, WA) since 2011. The EHR includes information from dispatch, the ePCR, and billing information. Responder impressions consist of 64 standardized options, which remained stable over the study period.^27^ Housing status is a mandatory field on ePCRs. Prehospital EMS responders are trained to assess the question “Is the patient homeless?” (yes vs no) on every LAFD-attended 9–1–1 EMS incident by asking the patient or, if the patient is unable or unwilling to respond, by applying their best judgment.

### Data Extraction

Data was extracted electronically from HealthEMS. We merged clinical data and EMS responder data using call number and booklet number, which are unique identifiers. Cases in which both the service date and call number were identical were dropped beyond the first instance. A sample of these cases were checked to ensure they were truly duplicates. We included cases in which “Narcan” or “Narcan nasal spray” were listed as medication that was administered during the incident. We stored all data was stored in a password-protected electronic spreadsheet (Microsoft Excel; Microsoft Corporation, Redmond, WA). The authors did not have access to the study population.

### Variable Definition and Modeling

To assess housing status, EMS responders asked each patient whether they were currently experiencing homelessness. If the patient was unable to answer, the EMS responder was instructed to use their best judgment based on their training.

We chose to define respiratory depression *a priori* as bradypnea with a respiratory rate of less than 12 breaths per minute, based on the LA County EMS Agency protocol and prior work evaluating prehospital naloxone administration.^28^^,^^29^ Although respiratory depression may also present as hypopnea, it is subjective and not reliably documented in the prehospital care report.

We extracted transport status from the disposition field on the ePCR. No transport was defined as an entry of “no transport/refused care,” “treated/no transport,” or “treated/no transport (AMA).” Transport was defined as an entry of “treated/transported.”

We modeled these variables as binary: housing status (currently unhoused yes/no, per EMS responder), identified as female (yes/no per EMS responder), respiratory depression (<12 breaths per minutes: yes/no) and transported (yes/no). The EMS responder’s impression and patient’s age were modeled as categorical.

The primary outcome was the prevalence of EMS administration of naloxone by housing status. Secondary outcomes included incidence of naloxone by patient characteristics, EMS responder’s impression, and transport status. We also examined whether disparate rates of naloxone administration remained robust after controlling for patient demographic and clinical characteristics in a regression model.

### Analysis

Our analyses used standard procedures for calculating descriptive statistics for the population of incidents. As our descriptive analyses were drawn from a complete compilation of calls rather than a sample, we followed the standard practice of excluding *P*-values for evaluating inferences about whether the sample statistics (eg, sample means) provided a reasonable estimate of the corresponding population parameters.

To understand whether the observed effect was explainable by core clinical or demographic factors, we performed a logistic regression analysis. Our model included age categories, gender, and clinical indication of respiratory depression because these factors were shown to have an effect in prior literature.^20^^,^^29^ The logistic regression formalized this, allowing us to test whether observed differences by PEH status were 1) reducible to clinical need or 2) reducible to other demographics. The logistic regressions do not provide a complete model of all possible explanations or establish causality, but rather help rule out alternative explanations of scientific and policy significance and to quantify important effects.

The descriptive statistics used the set of data for cases where naloxone was administered and for which we had data on housing status. Regression models provided information on the magnitude and direction of demographics and clinical effects on naloxone administration for the full population of EMS incidents. These models were used to describe associations in our data, not imply causality. Missing values were accounted for by list-wise deletion-- a common strategy for large datasets without high levels of missing data. All data were assembled, cleaned and modeled in STATA 14 (Stata Corp, College Station, TX). We produced figures using the ggplot2 package in R (The R Project for Statistical Computing, Vienna, Austria).

The study was reviewed and approved as exempt by the Institutional Review Board of the University of Southern California (HS-19-00472).

## RESULTS

**Figure 1.**
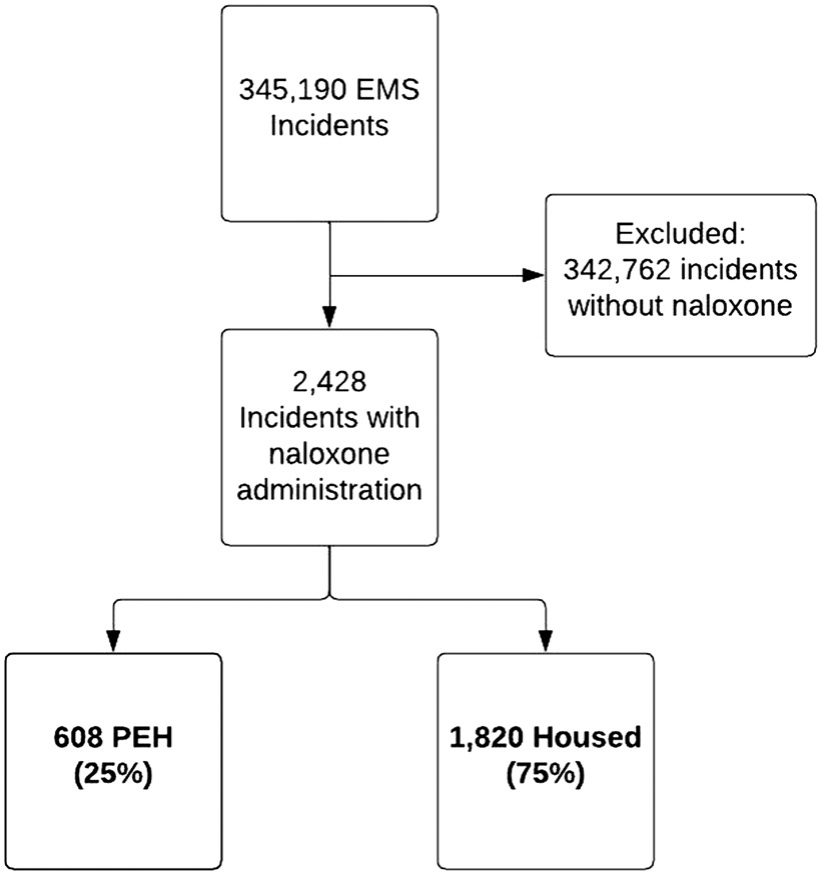
Study flow diagram. *EMS*, emergency medical services; *PEH*, persons experiencing homelessness.

**Figure 2.**
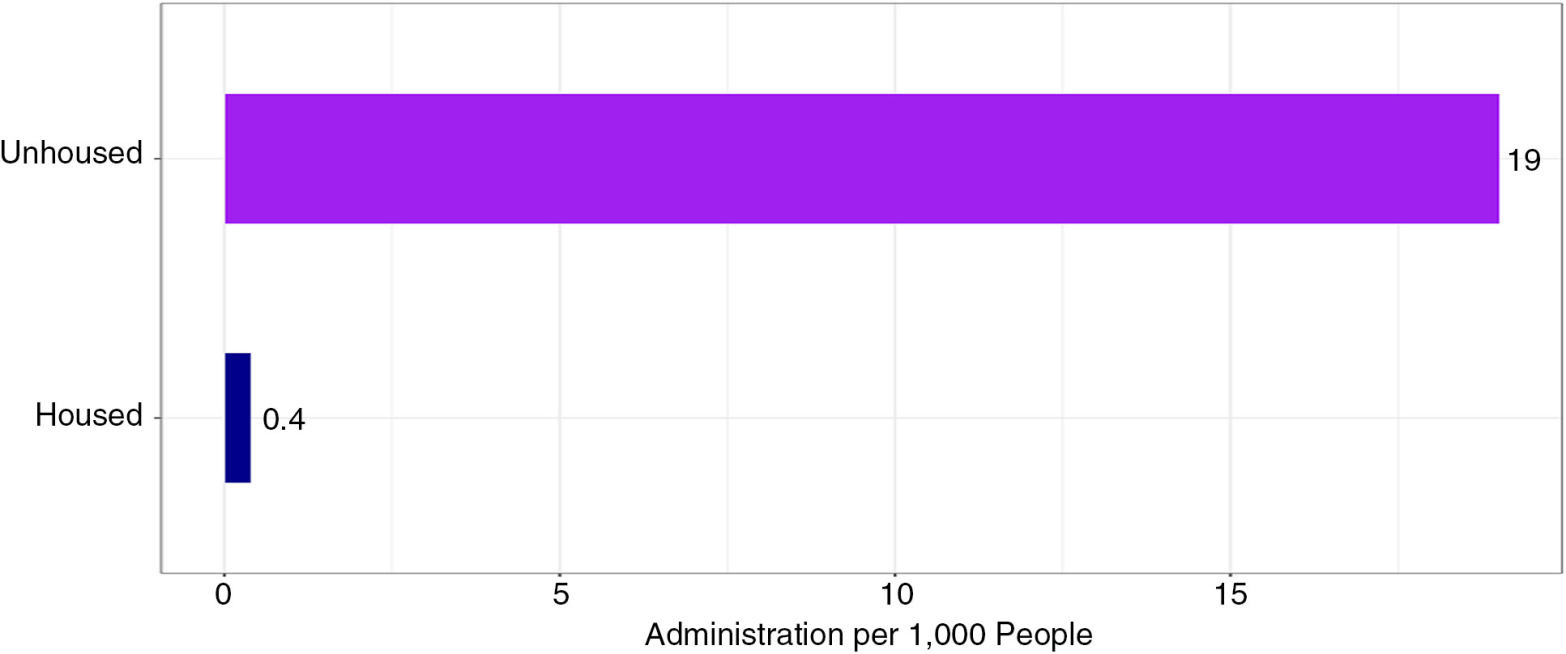
Naloxone administration rate by population.

Of the 345,190 unique, recorded 9–1–1 EMS incidents during the study period, 2,428 incidents met inclusion criteria (Figure 1). In the 2,428 incidents in which EMS administered naloxone, 608 (25%) incidents involved PEH, and 1,830 (75%) involved housed individuals. Incidents that resulted in naloxone administration occurred at a rate of 19 per 1,000 PEH compared to 0.4 per 1,000 housed individuals, or roughly 44 times the rate of housed individuals (Figure 2).

The study population had a mean age of 45 years (SD 19.4) and was 70.7% male. Of the patients who received EMS-administered naloxone, PEH were younger (mean 41.4 years [SD 14.1] vs 46.2 years [SD 20.7]) and more often male (81.9 vs 66.9%). The prevalence of patients who declined transport was higher for PEH than for housed individuals (17.3 vs 7.2%). The top three most common EMS responder impressions for which naloxone was administered were the same in both PEH and housed groups: overdose/poisoning/ingestion, altered level of consciousness and cardiac arrest (Table 1). Among those patients who received naloxone, a slightly greater proportion of the housed individuals were in cardiac arrest when compared to those experiencing homelessness (6.9 vs 4.3%). This does not change the primary finding or account for a substantial portion of the effect. Introducing the cardiac arrest variable in the model decreases the odds ratio [OR] from 2.38 to 2.35.

**Table 1.** Patient characteristics by housing status.

	All (N = 2,428)		PEH (n = 608)		Housed (n = 1,820)	
Mean age (years)	45 (SD 19.4)		41.4 (SD 14.1)		46.2 (SD 20.7)	
Median age (years)	53 (IQR 37)		47 (IQR 23)		54 (IQR 40)	
	n	%	n	%	N	%
Gender						
Female	712	29.3	110	18.1	602	33.1
Male	1,716	70.7	498	81.9	1218	66.9
Respiratory depression (RR < 12)	1,136	46.8	302	49.7%	834	45.8
Not transported	236	9.7	105	17.3%	131	7.2%
EMS professional impression^1^						
Overdose/poisoning/ingestion	1,373	56.3	399	66.2%	974	53.6%
Altered level of consciousness	695	28.5	153	25.4%	542	29.8%
Cardiac arrest	154	6.3	27	4.5%	127	7.0%

1 For EMS impression, eight charts were missing values (n = 2,420). There were no missing values for gender, respiratory depression, nor transport status.

*PEH,* persons experience homelessness; *IQR*, interquartile range; *RR*, respiratory rate.

**Figure 3.**
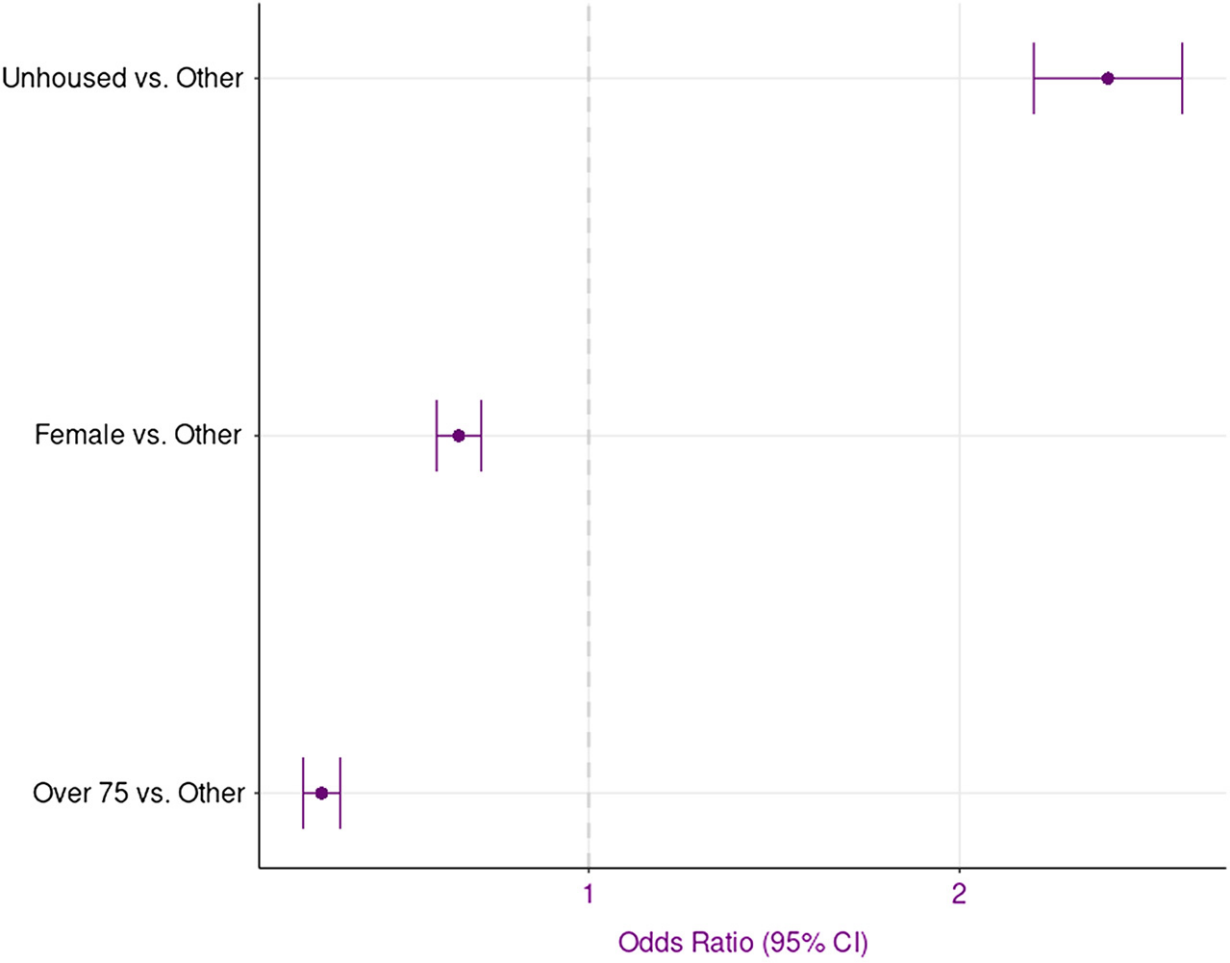
Adjusted odds of naloxone administration. *CI*, confidence interval.

The logistic regression shown in Table 2 demonstrates that even after accounting for key covariates (ie, age, respiratory depression, and gender), the odds of PEH being administered naloxone was 2.38 that of housed peers (95% confidence interval [CI] 2.15–2.64). This is visualized in Figure 3, which shows the post-adjustment odds of naloxone administration by group.

**Table 2.** Odds ratios for selected associations with naloxone administration.

Variable	OR (95% CI)	Adjusted OR (95% CI)
Homeless	2.61^*^ (2.38, 2.88)	2.38^*^ (2.15, 2.64)
Female		0.65^*^ (0.59, 0.71)
Respiratory depression		49.32^*^ (15.16, 53.87)
Age		
0–24		--
25–49		1.11 (0.98, 1.27)
50–74		0.54^*^ (0.47, 0/62)
>75		0.28^*^ (0.23, 0.33)

* P < .01.

*OR*, odds ratio; *CI*, confidence interval.

This data shows that even after adjusting for gender, age, and respiratory depression, 1) respiratory depression had the largest effect on whether naloxone was administered (OR 49.32, 95% CI 45.17–53.873) and 2) PEH had 2.38 higher odds of receiving EMS-administered naloxone relative to housed peers. This suggests that while administration mapped on to clinical factors on average, EMS responders administered naloxone at higher rates to PEH than to their housed counterparts irrespective of condition.

## DISCUSSION

In this study, PEH in the City of Los Angeles received EMS-administered naloxone at substantially higher rates than the housed population. While some of this may reflect need, PEH were still over two times more likely to receive the drug when all else was equal.

Secondarily, PEH who received naloxone tended to be younger and more often male when compared to their housed counterparts, although this did not explain the effect. This is consistent with prior studies documenting EMS utilization by PEH and the general differences in demographics between homeless and housed communites.^19^^,^^20^^,^^30^^,^^31^ However, it is notable that the mean age of our study population was younger than the average EMS user in the City of Los Angeles (45 vs 52 years). This difference is maintained for both the PEH and housed groups, 46.2 vs 52.6 years and 41.4 vs 46.1 years, respectively, suggesting that those receiving naloxone may be younger than the general population.^20^

Persons experiencing homelessness were more than two times as likely to refuse transport than their housed counterpart who received EMS-administered naloxone. However, prior studies in Los Angeles have demonstrated that overall, PEH were less likely to refuse transport against medical advice.^20^ Further, independent of housing status, refusal of transport was higher in patients receiving EMS-administered naloxone than overall refusal of treatment and/or transport against medical advice rate in Los Angeles during this study period.^20^ This highlights that there may be differences in clinical presentation, EMS care, patient-EMS interaction, and social situations associated with the management of presumed opioid overdose and OUD.

Our findings describe disproportionately high rates of administration of naloxone to PEH compared with their housed counterparts. The logistic regression suggests that experiencing homelessness is a predictor of naloxone administration net of other factors. This data highlights the discrepancy that persists even after controlling for age, gender, and respiratory status. However, this model does not distinguish whether this difference is due to a variation in clinical presentations or another factor that is leading EMS responders to administer naloxone when the patient’s medical emergency is related to an etiology other than opioid overdose. Future studies are needed to understand the differences in care provided by EMS to PEH vs housed individuals and to evaluate patient outcome data. These findings can help drive future EMS education and field interventions, and potentially help develop specialized prehospital programs that focus on opioid overdose and risk reduction in this vulnerable population.

Although this study does not address patient outcomes, we must discuss the potential clinical impact of higher rates of naloxone administration on patient outcomes. Naloxone is a relatively safe drug. However, there are risks associated with administering high doses of naloxone given the dose-dependent relationship between naloxone and pulmonary edema. A recent prehospital study demonstrated higher rates of pulmonary complications, such as pulmonary edema and need for ventilatory support, in cases in which higher doses of out-of-hospital naloxone were administered.^32^ Further, administering naloxone in cases where patients have OUD, but opioid overdose is not the etiology of their symptoms, may unnecessarily precipitate acute opioid withdrawal, vomiting, and aspiration. Finally, administering excessive or unnecessary naloxone detracts from EMS responders’ ability to critically assess the situation and treat the primary medical emergency. Thus, PEH are at potentially higher risk for poor outcomes given the higher rates of EMS-administered naloxone. Further studies are needed that incorporate patient outcome as well as patient and EMS responders’ experiences to elucidate potential biases in care.

Further, this study identifies a potential target for patient-centered interventions. Prior studies have suggested that by increasing access to naloxone, opioid overdose mortality can be decreased.^33^^–^^35^ However, in California only 6% of local EMS agencies had EMS-based outreach programs and 9% oversaw naloxone distribution.^36^ Given that EMS may be the first, or only, medical care that an individual receives, this interaction provides the potential for OUD-related care, medication-assisted therapy, naloxone administration, and/or linkage to care. The EMS agency is in a unique position of having situational awareness and regular contact with PEH, which can be leveraged to address the needs of this at-risk population. Through prehospital interventions and novel care pathways, there may be opportunities to improve patient outcomes in a more cost-effective and culturally acceptable manner.

Finally, this study is the first step in describing the disparities of EMS-administered naloxone by housing status. Persons experiencing homelessness were administered naloxone at a substantially higher rate than the population as a whole (19 vs 0.4 per 1,000 members of the population). Much of this reflects differences in need. However, our analyses show that unhoused individuals remained more than twice as likely as housed peers to be administered naloxone even after adjusting for clinical and demographic factors. Future research will be necessary to determine the cause and scope of these patterns.

## LIMITATIONS

Because this was a retrospective observational study it has limitations inherent to study design and clinical documentation. The available data is subject to reporting errors and missing data points. Nor were we able to assess temporality of the respiratory rate in relation to the patient receiving naloxone, since the timing of vitals and interventions were documented by the EMS responders in retrospect and, therefore, were not precise enough. Additionally, it is possible that additional clinical characteristics other than bradypnea impact an EMS responder’s decision to administer naloxone. Given the variability in documentation, assessment of neurologic status and airway compromise were not included in this analysis but may have impacted whether a patient received naloxone. Further, this study relies upon observation data and was not designed to establish causality. While the effect of homelessness was not eliminated in models adjusting for core clinical indications or demographics (age, gender), it is possible that the effect is reducible to latent variables or confounders that are absent from our data.

Further, homelessness is a complex and sometimes transient issue. The EMS responders were responsible for documenting the patients’ housing status. Given the binary option in the ePCR and the training provided, it is possible that patients’ housing status could potentially have been inaccurately coded in either direction. The decision to document housing status as homeless may be biased by appearance, environment, presence of paraphernalia, and even the use of naloxone itself.

Additionally, this study only accounts for naloxone administration by EMS and does not include naloxone administered by bystanders or other first responders, such as law enforcement or street medicine teams. Further, although this study captures all patients who were administered naloxone by EMS, it does not capture all patients who may have had opioid or substance use disorders. Given the existing body of literature that suggests a high incidence of SUD, including OUD, in the unhoused population, it is likely that an even larger number of EMS patients who are homeless may be experiencing an emergency related to OUD/SUD even when not explicitly labeled with an EMS responder’s impression related to overdose or intoxication or administered naloxone. While this cannot be further extrapolated due to limitations in the ePCR data, this relationship has previously been described in the emergency medicine literature.^30^ Thus, an even larger number of patients could potentially benefit from outreach programs or other interventions.

Finally, the study was conducted in a single city. As a city with one of the largest populations of PEH, Los Angeles was used as a lens to evaluate the evolving situation at the intersection of the opioid epidemic, the housing crisis, and EMS. While Los Angeles may have unique characteristics, prior studies suggest that the demographics of its homeless population are similar to other major US cities.^31^ Given national trends, Los Angeles may serve as a bellwether for other metropolitan areas in the US.

## CONCLUSION

Persons experiencing homelessness in the City of Los Angeles received EMS-administered naloxone at higher rates than their housed counterparts, even when accounting for differences in age, gender, and respiratory depression. Future research is needed to validate these findings in other settings and to understand this difference in administration rates, characterize patient outcomes, and identify potential targets for alternative care pathways for patients confronting homelessness and opioid use disorder.
